# An overview of current research on the modulation of NLRP3 inflammasome by traditional Chinese medicine to combat acute pancreatitis

**DOI:** 10.3389/fmolb.2025.1634132

**Published:** 2025-07-16

**Authors:** Xiongjie He, Jia Xia, Qi Chen, Zhaozhao Huang, Juanjuan Lu, Yisong Ren

**Affiliations:** ^1^ Department of Critical Care Medicine, Chengdu Pidu District Hospital of Traditional Chinese Medicine, Chengdu, Sichuan, China; ^2^ Department of Endocrinology, Chengdu Pidu District Hospital of Traditional Chinese Medicine, Chengdu, Sichuan, China

**Keywords:** acute pancreatitis, traditional Chinese medicine, NLRP3, inflammation, antiinflammatory

## Abstract

Acute pancreatitis (AP), a life-threatening gastrointestinal emergency, is characterized by acute onset, rapid clinical deterioration, and high mortality rates, imposing profound long-term health burdens and socioeconomic costs on patients and healthcare systems. Current therapeutic strategies focus on supportive care, as no curative therapies exist to halt AP progression. Traditional Chinese medicine (TCM), with its multi-target, multi-component, and multi-pathway pharmacological properties, has emerged as a promising therapeutic drug against inflammation-driven pathologies, including AP. This review systematically discussed the assembly, activation, and pathogenic contributions of the NOD-like receptor family pyrin domain-containing 3 (NLRP3) inflammasome in AP pathogenesis. Mechanistically, NLRP3 activation exacerbated pancreatic injury through caspase-1-dependent maturation of interleukin-1β (IL-1β) and gasdermin D (GSDMD)-mediated pyroptosis, perpetuating systemic inflammation. We systematically summarized the research progress of TCM in the treatment of AP by reducing pancreatic necrosis, neutrophil infiltration, and intestinal barrier dysfunction through targeting NLRP3 inflammasome, as well as its clinical evidence. Collectively, this review highlights the translational potential of TCM as an adjunctive therapy for AP through NLRP3 inflammasome inhibition, offering mechanistic insights and evidence-based support for its integration into integrative medicine strategies.

## Graphical Abstract

**Figure:**
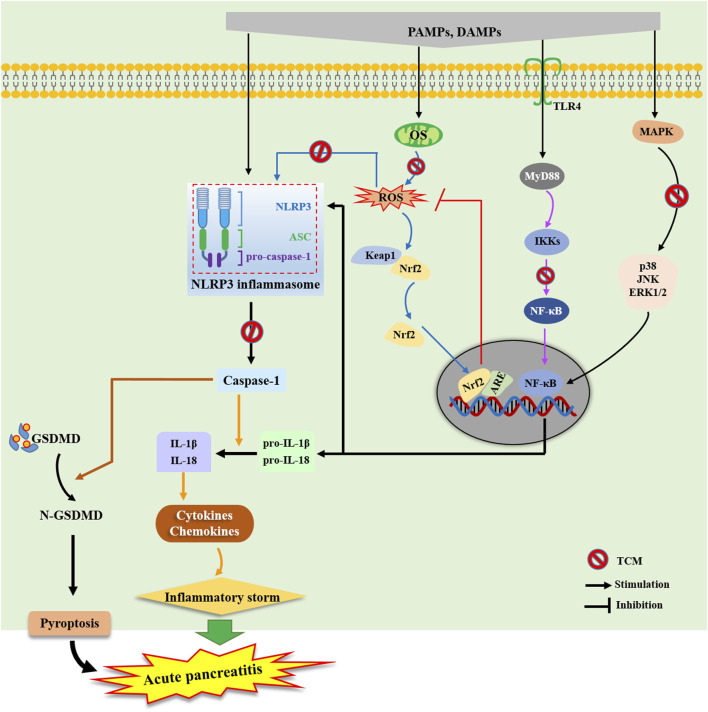
The functional role and mechanisms of TCM in the prevention and treatment of AP by inactivation of NLRP3 inflammasome. TCM, traditional Chinese medicine; OS, oxidative stress; TLR4, toll-like receptor 4; MAPK, mitogen-activated protein kinase; JNK, c-Jun N-terminal kinase; ERK, extracellular signal-regulated kinase; Nrf2, nuclear factor-erythroid factor 2-related factor 2; ARE, antioxidant response element; Keap1, Kelch-like ECH-associated protein 1; AP, acute pancreatitis.

## 1 Introduction

Acute pancreatitis (AP) is a life-threatening inflammatory disorder characterized by premature activation of pancreatic enzymes, leading to autodigestion, edema, hemorrhage, and necrosis of pancreatic tissues (Mederos et al., 2021). Epidemiological data indicated that approximately 20% of patients with AP developed severe AP, which was characterized by dysregulated cytokine storms that exacerbate pancreatic damage and precipitate multi-organ failure (e.g., pulmonary insufficiency, intestinal barrier dysfunction) (Garg and Singh, 2019). Global health statistics revealed that the global incidence and death of AP increased by 2.75 million and 122,416 in 2021 (Iannuzzi et al., 2022), which undoubtedly imposed substantial socioeconomic burdens on patients, families, and healthcare systems. Current management paradigms remain predominantly supportive, prioritizing aggressive fluid resuscitation, opioid-sparing analgesia, and enteral nutrition to mitigate systemic complications (Petrov and Yadav, 2019). Invasive procedures were reserved exclusively for managing refractory complications, such as infected necrotizing pancreatitis or persistent pseudocyst-related symptoms (Gardner et al., 2020). These clinical challenges underscored the urgent need to unravel AP pathogenesis and developed effective preventive and therapeutic strategies.

A critical driver of AP pathogenesis was the dysregulated activation of the nucleotide oligomerization domain (NOD)-like receptor family pyrin domain-containing 3 (NLRP3) inflammasome, a cytosolic multiprotein complex that orchestrates caspase-1-mediated maturation of pro-inflammatory cytokines interleukin-1β (IL-1β) and IL-18, as well as gasdermin D (GSDMD)-dependent pyroptotic cell death (Ferrero-Andrés et al., 2020). NLRP3 inflammasome was a group of multi-protein complexes that participate in innate immunity processes through the activation of pro-inflammatory caspases (Martinon et al., 2002). In AP, damage-associated molecular patterns (DAMPs) released from necrotic acinar cells, such as mitochondrial DNA, extracellular ATP, and reactive oxygen species (ROS), which activated NLRP3 inflammasome and resulted in perpetuating a vicious cycle of inflammation, pancreatic necrosis, and systemic inflammatory response syndrome (Papantoniou et al., 2024). This inflammatory cascade was further amplified by infiltrating immune cells, including macrophages and neutrophils, which secreted additional inflammatory cytokines and chemokines, exacerbating tissue injury (Wan et al., 2020). Numerous studies have proved that activation of the NLRP3 inflammasome was associated with the severity of AP (Jia et al., 2020; Sendler et al., 2020). Preclinical studies have demonstrated that targeted inactivation of NLRP3 inflammasome [such as indomethacin (Lu G. et al., 2017), INT-777 (Li B. et al., 2018), apocynin (Jin et al., 2019), T-614 (Hou et al., 2019), and MCC950 (Sendler et al., 2020)] alleviated AP progression and associated organ injury by suppressing inflammation and pancreatic acinar cell apoptosis. Meanwhile, the inflammatory cytokines IL-18 and IL-1β may serve as the markers of the severity of AP patients (Janiak et al., 2015). Current therapeutic strategies, however, remain largely palliative, underscoring the urgent need for mechanistically targeted interventions to disrupt NLRP3-driven AP progression.

Traditional Chinese Medicine (TCM) is one of the ancient and most accepted alternative medicinal systems in the world for the treatment of health ailments, especially when Western medicine is not very effective (Luo et al., 2019). For hundreds of years, medicinal herbs have been used with apparent safety and efficacy for alleviating and treating AP in China. TCM, with its multi-component, multi-target, and multi-pathway pharmacological profiles, has emerged as a promising approach to modulating NLRP3 inflammasome activity in inflammation-associated diseases (Xue et al., 2023). Unlike synthetic inhibitors such as MCC950, a potent NLRP3 antagonist with clinical limitations due to off-target effects and pharmacokinetic challenges, TCM exerts pleiotropic pharmacological effects with low toxicity, including anti-inflammatory, antioxidant, antitumor, and immunomodulatory properties (Li and Zhang, 2013). For instance, *Chaihuang Qingfu pill* prevented severe AP-induced lung injury by inhibiting NLRP3-mediated macrophage pyroptosis (Xiao et al., 2025). Chlorogenic acid attenuated the development of severe AP by inhibiting NLRP3 inflammation activation and activating the Nrf2/HO-1 pathway (Ye et al., 2025). *Psidium guajava* flavonoids exerted a protective role in severe AP by inactivation of NLRP3 inflammasome (Zhang G. et al., 2021). Of note, clinical studies have also confirmed that TCM formulas were given to slow down the progression of AP (Zhang et al., 2008), as well as improve immune function (Jiang et al., 2016) and gastrointestinal function (Miao et al., 2018). Moreover, combined treatment of TCM and Western medicine contributed to reducing inflammation and improving immune dysfunction compared with Western medicine alone for patients with AP (Liu et al., 2011; Chen et al., 2021). Deng et al. (2024) showed that integrated TCM and Western medicine treatment reduced the risks of mortality and organ failure and achieved better economic effectiveness in patients with AP than Western medicine alone treatment. These results indicated that TCM prescriptions, monomers, and extracts possessed an inhibitory effect on NLRP3 inflammation, which could benefit the treatment of AP. However, there is still a lack of comprehensive review on TCM regulation of NLRP3 inflammasome-associated pathways in the treatment of AP.

Herein, we discussed the NLRP3 and its functional role in the development and progression of AP. Moreover, we summarized the therapeutic effect of TCM (prescriptions, extracts, and monomer compounds) on AP by targeting NLRP3 inflammasome-associated pathways. Furthermore, we analyzed the efficacy and safety of TCM for the treatment of AP in clinical trials, and discussed their challenges and future development directions.

## 2 Research methodology

This review article was conducted using electronic databases such as PubMed, Google Scholar, Springer Link, Science Direct, Cochrane Library, Embase, Web of Science, and Scopus. All published data till the year 2025 have been taken into consideration. The following search keywords were used in the search of materials for this study: “acute pancreatitis”, “NLRP3 inflammasome”, “inflammasome”, “medicinal plants”, “TCM prescription/decoction/formula”, “herbal extract”, “TCM extract”, “bioactive compounds”, “active ingredients”, “polyphenols”, “flavonoids”, “alkaloids”, “terpenes”, “anthraquinones”, “shikonin”, “polysaccharide”, “biological activity”, “pharmacological activities”, and other similar keywords in combination with words such as traditional Chinese medicine, Clinical trials, botanical description, toxicity, human health, and nutritional composition. All articles addressing these principal keywords were considered when available in the English language, and in peer-reviewed journals, whether published as review or research articles. Papers were reviewed in their entirety if their abstract mentioned that the article presented any potential relevance to the inclusion criteria. Articles were excluded based on title, abstract, or full text because of their lack of pertinence to the issue concerned. Articles were excluded if they were letters, comments, or not available for access to full article.

## 3 Overview of NLRP3 inflammasome

Innate immunity serves as the host’s primary defense barrier, wherein pattern recognition receptors (PRRs) on immune cells detect pathogen-associated molecular patterns (PAMPs), such as viral nucleic acids, bacterial lipopolysaccharides, and flagellin, or endogenous DAMPs released from damaged or dying cells. This recognition initiates innate immune responses and activates downstream inflammatory pathways to eliminate microbial infections and promote tissue repair (Kelley et al., 2019; Fu and Wu, 2023). In 2002, a novel class of PRR termed the inflammasome was identified as a critical signal transduction platform in innate immunity (Martinon et al., 2002). Certain members of the NOD-like receptor (NLR) family assemble into multiprotein complexes, forming a subset of inflammasomes (Toldo et al., 2022). To date, at least 22 inflammasome subtypes have been characterized, with the NLRP3 inflammasome being the most extensively studied (Zhuang et al., 2021). Under physiological conditions, NLRP3 inflammasome activation is essential for host defense against pathogens and homeostatic maintenance. However, its dysregulated activation drives excessive inflammatory responses and host tissue damage, contributing to autoimmune disorders such as AP (Papantoniou et al., 2024).

### 3.1 Structure of NLRP3 inflammasome

The NLRP3 inflammasome is composed of a sensor (NLRP3), an adaptor (ASC; also known as PYCARD), and an effector (Caspase-1) (Huang Y. et al., 2021). NLRP3, a trimeric protein, encompasses three functional domains: (1) the C-terminal leucine-rich repeat (LRR), which is crucial for ligand sensing; (2) the central nucleotide-binding and oligomerization (NACHT) domain, and (3) the amino-terminal pyrin domain (PYD) that mediates protein-protein interactions (Unterberger et al., 2021). Upon stimulation by PAMPs or DAMPs, NLRP3 oligomerizes homotypically through its NACHT domain. The oligomerized NLRP3 then recruits the apoptosis-associated speck-like protein containing a CARD (ASC) via PYD-PYD interactions. Subsequently, ASC facilitates the assembly of the NLRP3-ASC-pro-caspase-1 complex (the canonical NLRP3 inflammasome) by binding pro-caspase-1 through CARD-CARD interactions. Activation of the NLRP3 inflammasome triggers autoproteolytic cleavage of pro-caspase-1 into enzymatically active caspase-1, which catalyzes the maturation of pro-inflammatory cytokines IL-1β and IL-18 (Lechtenberg et al., 2014). These cytokines, in turn, amplify immune responses through downstream signaling cascades. Concurrently, active caspase-1 cleaves GSDMD to generate N-terminal fragments that form plasma membrane pores, inducing a lytic programmed cell death pathway associated with inflammatory cytokine release (Li L. et al., 2024).

### 3.2 Mechanism of NLRP3 inflammasome activation

**FIGURE 1:**
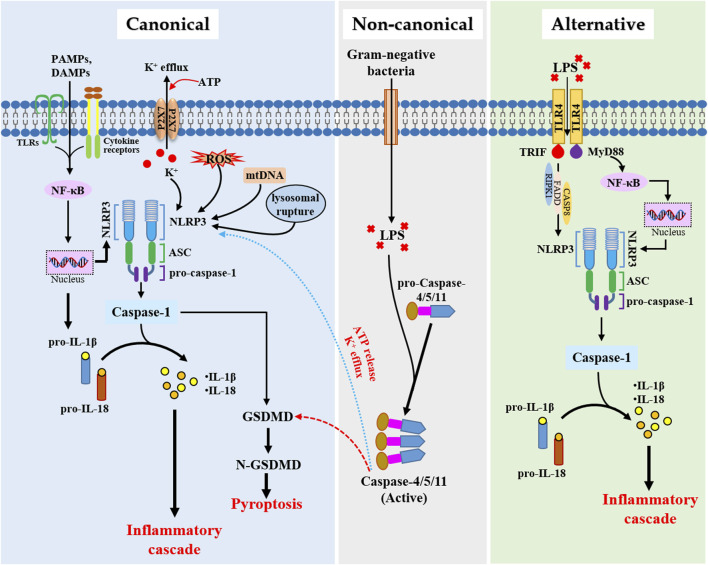
Mechanisms of NLRP3 inflammasome activation pathways. PAMPs, pathogen-associated molecular patterns; DAMPs, damage-associated molecular patterns; NF-κB, nuclear factor kappa-B; NLRP3, NOD-like receptor family pyrin domain-containing 3; ASC, apoptosis-associated speck-like protein containing a caspase recruitment domain; ROS, reactive oxygen species; mtDNA, mitochondrial DNA; IL, interleukin; GSDMD, gasdermin D; LPS, lipopolysaccharide; TRIF, TIR-domain-containing adapter-inducing interferon-β; RIPK1, receptor-interacting protein kinase 1; FADD, FAS-associated death domain; CASP8, caspase-8.

Currently, NLRP3 inflammasome activation pathways are broadly categorized into canonical, noncanonical, and alternative pathways (Figure 1), with classification criteria predicated on stimulus specificity (e.g., PAMP vs. damage-associated signals) and cell type (e.g., immune vs. non-immune cell types) (Seoane et al., 2020). The canonical NLRP3 inflammasome activation requires two sequential priming and activation steps. The priming phase is initiated when DAMPs or PAMPs engage receptors such as toll-like receptors (TLRs) or cytokine receptors, triggering the NF-κB pathway to increase mRNA levels of NLRP3, pro-IL-1β, and pro-IL-18 (Zahid et al., 2019). Beyond transcriptional priming, this phase also orchestrates NLRP3 post-translational modifications (PTMs), including phosphorylation, ubiquitination, and SUMOylation, which play an important role in NLRP3 activation (Paik et al., 2021; Xu et al., 2022). The activation phase, the second step in the canonical pathway, is driven by diverse stimuli such as K^+^ efflux, ROS overproduction, lysosomal rupture, mitochondrial DNA (mtDNA) leakage, and organelle dysfunction (e.g., mitochondria, Golgi apparatus, endoplasmic reticulum) (Akbal et al., 2022). These events promoted NLRP3 inflammasome activation. In the noncanonical pathway, NLRP3 inflammasome activation is induced by lipopolysaccharide (LPS) internalization into the cytosol by transfection or infection (Santos et al., 2018), which can be recognized by caspase-11 (the mouse homolog of human caspase-4/5). Then, activated caspase 4/5/11 causes pyroptosis by cleaving GSDMD, and also triggers the assembly of NLRP3 inflammasome. Cross-talk between non-canonical and canonical inflammasome activation pathways, such as the activation of pannexin-1 by caspase-11 and subsequent release of ATP and activation of P2X7R to induce K^+^ efflux and thus canonical NLRP3 assembly, has also been suggested (Downs et al., 2020). Distinct from the above pathways, the alternative pathway of NLRP3 inflammasome activation bypasses pyroptosis, ASC polymerization, and K^+^ efflux, which has been exclusively characterized in primary human and porcine monocytes. Mechanistically, LPS directly activated NLRP3 inflammasome through the TLR4-TRIF-MyD88-RIPK1-FADD-CASP8 axis without eliciting pyroptotic cell death (He et al., 2016).

## 4 NLRP3 inflammasome in the pathogenesis of AP

AP is an inflammatory disorder characterized by excessive activation of pancreatic enzymes due to diverse etiological factors, leading to autodigestion, edema, hemorrhage, and necrosis of pancreatic parenchyma and adjacent tissues. A clinical study has confirmed elevated expression of NLRP3 inflammasome components in serum samples from pancreatitis patients compared to healthy controls, with NLRP3 activation correlating positively with disease severity (Algaba-Chueca et al., 2017). Sendler et al. (2020) reported that increased levels of proinflammatory cytokines (IL-1β and IL-18) and ASC were detected in serum samples from patients with severe AP. Hoque et al. (2011) further identified that NLRP3 inflammasome activation was a driver of disease progression through amplification of initial inflammatory cascades. Functionally, NLRP3 inflammasome were commonly activated by DAMPs (e.g., HMGB1 and HSP70) or PAMPs stimulation, which further promoted pancreatic inflammation and tissue injury, eventually advancing AP (Hoque et al., 2011). Meanwhile, LPS-stimulated-caspase-4/5/11 further triggers GSDMD driven pancreatic cell death and tissue injury, leading to the course of the pathogenesis of AP (Yi, 2020). In addition, ROS-mediated TXNIP and the activation of P2X7 trigger the NLRP3 inflammasome (Zhang et al., 2017). In mouse models of AP, NLRP3 deficiency ameliorated pancreatic inflammation and associated complications by reducing neutrophil infiltration (Fu et al., 2018). Another study showed that NLRP3 inflammasome activation promoted lung dysfunction by triggering alveolar macrophage pyroptosis in pancreatitis progression (Wu et al., 2020). In a recent study, pharmacological inhibition of NLRP3 inflammasome by MCC950 improved pathological damage and reduced inflammatory response in experimental pancreatitis (Shen et al., 2022). Mechanistically, the inflammation mediated by the Caspase-1/NLRP3, TLR4/NLRP3, MAPK/NF-κB/NLRP3, and Nrf2/HO-1/NLRP3 pathways have been implicated in the pathogenesis and progression of AP (Papantoniou et al., 2024), highlighting their potential as therapeutic targets. Collectively, these findings indicated that inhibition of NLRP3 inflammation may be a promising therapeutic strategy for AP management.

## 5 TCM used to relieve AP by targeting NLRP3

In Chinese medicine (CM), AP is classified under abdominal pain (*Futong*), spleen-heart pain (*Pi Xintong*), and pancreatic inflammation (*Yi Dan*) (Li et al., 2019a). Etiological factors in CM theory include gallstone obstruction, dietary irregularities (e.g., excessive greasy food), emotional disturbances (*Gan Yu*), trauma, and invasion of exogenous pathogens (*Liu Yin*) (Wu, 2002), all of which may exacerbate the progression of AP. Pathophysiologically, it manifests as root deficiency (*Ben Xu*) with branch excess (*Biao Shi*), predominantly presenting as interior excess-heat syndrome (*Li Shi Re Zheng*) (Mao et al., 2003). CM therapeutic strategies emphasize heat-clearing and bowel-unblocking methods (*Qing Re Tong Fu Fa*), integrating root-cause treatment (*Zhi Ben*) with symptomatic management (*Zhi Biao*) (Li et al., 2019a). Emerging pharmacological evidence has demonstrated that TCM prescription, extract, and active ingredient mitigated AP progression by suppressing NLRP3 inflammasome activation and downstream pyroptosis pathways (Jiang et al., 2021; Zeng et al., 2024; An et al., 2025). Moreover, the functional role of TCM in AP by regulating NLRP3 inflammation is summarized in Table 1 and 2.

**TABLE 1:** Summary of TCM prescriptions and extracts in the prevention and treatment of AP by modulating inflammation in the last 5 years.

Prescription	Composition (botanical name)	Model and dose	Effects	Ref.
*Da Cheng Qi decoction*	*Rheum palmatum* L. 12 g, Na_2_SO_4_⋅10H_2_O 7.5 g, *Fructus Aurantii Immaturus* 9 g, *Houpoea officinalis* 9 g	Model ①Carulein plus LPS-induced SAP ②Carulein plus LPS-induced AR42J cells Dose ①7 g/kg BW ②10 mg/L	↓Levels of amylase, LDH, IL-6, IL-1β, TNF-α, HMGB1, ROS, NOX2 ↑GPX4 expression ↓Cell ferroptosis	Chen et al. (2025)
*Qingyi decoction*	*Radix et Rhizoma Rhei* 15 g, *Bupleuri Radix* 15 g, *Aucklandiae Radix* 10 g, *Corydalis Rhizoma* 10 g, *Paeoniae Radix Alba* 15 g, *Scutellariae Radix* 10 g, *Coptidis Rhizoma* 10 g	Model: Caerulein plus LPS-induced SAP-ALI Dose: 6, 12, and 24 g/kg BW	↓Cytokine storm, pancreas edema, and serum amylase ↓Levels of TNF-α, IL-1β, and IL-6 ↓NLRP3/Caspase-1/GSDMD pathway	An et al. (2025)
*Dachaihu decoction*	*Bupleurum chinense* DC. 15 g, *Scutellaria baicalensis* Georgi 9 g, *Citrus × aurantium* L. 9 g, *Paeonia lactiflora Pall.* 9 g, *Pinellia pedatisecta* Schott 9 g, *Rheum palmatum* L. 6 g, *Zingiber officinale Roscoe* 15 g, *Ziziphus abyssinica Hochst* 20 g	Model: Caerulein-induced AP Dose: 5.5, 11, and 22 g/kg BW	↓The pathological scores for edema, inflammatory infiltration, fibrosis, and acinar atrophy ↓Expression of COL1A1, α-SMA, IL-6, MCP-1, and TNF-α ↓MAPK pathway	Li et al. (2025)
*Qingjie Huagong decoction*	*Bupleuri radix* 12 g, *Scutellariae radix* 10 g, *Magnoliae officinalis cortex* 8 g, *Salviae miltiorrhizae radix et rhizoma* 9 g, *Rhei radix et rhizoma* 6 g, *Aurantii fructus immaturus* 10 g, and *Glycyrrhizae radix et rhizome* 5 g	Model: Caerulein-induced AP Dose: 6.5, 13, and 26 g/kg BW	↓Levels of IL-1β, IL-6, IL-8, IL-18, and TNF-α ↓Expression of NLRP3, TLR4, MyD88, NF-κBp65	Feng et al. (2024)
*Rhizoma Alismatis decoction*	*Alisma orientalis* 5 g and *Atractylodes macrocephala* 2 g	Model: Caerulein-induced AP Dose: 4 and 36 g/kg BW	↓Pancreas injury ↓Levels of IL-6, TNF-α, IL-1β, and IL-18 ↓Mitochondrial oxidative damage and dysfunction ↓Apoptosis of acinar cells ↓NLRP3 inflammasome activation	Zhang et al. (2024b)
*Qing Xia Jie Yi formula*	*Rheum palmetu m* L. 15 g, *Citrus × aurantium* L. 12 g, *Sargentodoxa cuneata (Oliv.)* 30 g, *Gardenia jasminoides* J.Ellis 9 g, *Bupleurum marginatum* Wall. ex DC. 9 g, *Corydalis yanhusuo* 12 g, *Salvia miltiorrhiza Bunge* 15 g, *Paeonia lactiflora Pall.* 15 g	Model: Caerulein-induced AP Dose: 4.8 mg/g for 3 times at 3 h, 5 h and 7 h after the first caerulein injection	↓Pancreas injury ↓The infiltration of F4/80^+^ macrophage and Ly6G^+^ neutrophils in the pancreas ↓The serum levels of TNF-α, IL1β, and IL6 ↓M1 macrophages polarization	Han et al. (2024)
*Qingyi decoction*	*Rhubarb* 20 g, *Radix Bupleuri* 15 g, *Radix Aucklandiae* 15 g, *Paeoniae Radix Alba* 15 g, *Natrii Sulfas* 10 g, *Rhizome Corydalis* 15 g, *Gardenia jasminoides* 15 g and *Scutellaria baicalensis* Georgi 12 g	Model: Caerulein plus LPS-induced SAP-ALI Dose: 7.6 g/kg BW	↓Levels of MPO, α-amylase, IL-1β, IL-6, and TNF-α ↑The relative abundance of SCFAs-producing bacteria ↓Intestinal permeability ↓AMPK/NF-κB/NLRP3 pathway	Wang et al. (2023)
*Chaiqin chengqi decoction*	*Rheum palmetu m* L. 20 g, *Gardeniajasminoides* J.Ellis 20 g, *Bupleurum marginatum* Wall. ex DC. 15 g, *Magnolia officinalis* Rehder and E.H.Wilson 15 g*, Citrus × aurantium L.* 15 g, *Scutellaria baicalensis* Georgi 15 g*, Artemisia capillaris* Thunb. 15 g, Sodium sulfate 20 g	Model: Caerulein-induced AP Dose: 1, 5, and 10 g/kg BW	↓Pancreatic injury and systemic inflammation ↓Serum amylase, serum lipase, MPO, and F4/80 ↓GSDMD mediated pyroptosis ↓Levels of NLRP3, GSDMD, and cleaved caspase-1	Cao et al. (2024)
		Model: Caerulein-induced AP Dose: 5, 10, and 20 g/kg BW	↓Pancreatic injury and systemic inflammation ↓Necrotic cell death ↓TLR4/NLRP3 pathway	Wen et al. (2020)
*Xiaochaihu decoction*	*Radix Bupleuri* 24 g, *Arum ternatum* Thunb 9 g, *Scutellariae Radix* 9 g, *Zingiber officinale Roscoe* 9 g, *Panax ginseng* C. A. Mey. 9 g, *licorice* 9 g, *Jujubae Fructus* 12 g	Model: LPS-induced AR42J cells Dose: 12.5, 25, 50, and 100 μM	↑Cell viability ↓Levels of IL-1β, IL-6, and TNF-α ↓MAPK3 and TP53	Zhan et al. (2021)
*Yue-Bi-Tang*	*Ephedrae herba* 18 g, *Zingiber officinale Roscoe* 9 g, CaSO_4_·2H_2_O 24 g, *Ziziphus jujuba* Mill. 9 g, *Glycyrrhiza uralensis* Fisch. 6 g	Model: NaT-induced SAP Dose: 5.63 g/kg BW	↑Serum levels of IL-10 and ↓TNF-α ↓Necrosis and interstitial edema ↓Contents of MDA	Hu et al. (2020)

Extract	Model and dose	Effects	Ref.
Free total rhubarb anthraquinones	Model: NaT-induced SAP Dose: 22.5, 45, and 90 mg/kg BW	↓Damaged intestine and pancreas ↑Expression of intestinal epithelial junction proteins ↓Levels of DAO, IL-1β, IL-18, HMGB1, and LDH ↓NLRP3-Caspase-1-GSDMD and TLR-4-NF-κB pathways	Zeng et al. (2024)
*Psidium guajava* flavonoids	Model: Caerulein-induced AP Dose: 0.186 and 0.372 g/kg BW	↓The degrees of acinar atrophy, fibrosis, and inflammatory cell infiltrate ↓Expression of Collagen Ⅰ and Ⅲ and α-SMA ↓Expression of IL-1β and IL-18 ↓NLRP3/Caspase-1	Zhang et al. (2021a)
Total flavonoids of *Chrysanthemum indicum* L	Model: Caerulein-induced AP Dose: 300 mg/kg BW	↓Serum amylase, water content of pancreatic tissues, and MPO ↓Levels of IL-6, TNF-α, IL-1β, COX-2, MCP-1, and CXCL16 ↓NF-κB pathway	Yang et al. (2023a)
Aqueous extraction from dachengqi formula	Model: Caerulein-induced AP Dose: 0.6, 1.2, and 2.4 g/kg BW	↓Pancreas edema inflammation, and necrosis ↓MPO and inflammatory cytokines (IL-1β, IL-6, and TNF-α) ↓p38MAPK/NF-κB pathway	Ma et al. (2020)

Note: LPS, lipopolysaccharide; SAP-ALI, severe acute pancreatitis (AP)-associated acute lung injury (ALI); MPO, myeloperoxidase; BW, body weight; MDA, malondialdehyde; LDH, lactate dehydrogenase; IL, interleukin; TNF-α, tumor necrosis factor alpha; HMGB1, high mobility group box 1; ROS, reactive oxygen species; NOX2, NADPH, oxidase 2; GPX4, glutathione peroxidase 4; NLRP3, NOD-like receptor family pyrin domain-containing 3; GSDMD, gasdermin D; MCP-1, monocyte chemoattractant protein-1; α-SMA, alpha -smooth muscle actin; MAPK, mitogen-activated protein kinase; TLR4, toll-like receptor 4; MyD88, myeloid differentiation primary response 88; NF-κB, nuclear factor-kappaB; SCFAs, short-chain fatty acids; AMPK, AMP-activated protein kinase; DAO, diamine oxidase; CXCL16, CXC, motif chemokine ligand 16.

**TABLE 2:** Summary of active components of TCM in the prevention and treatment of AP in the last 5 years.

Compound	Model and dose	Effect	Ref.
Chlorogenic acid	Model ①Caerulein plus LPS-induced SAP ②Cerulein-treated AR42J cells Dose ①20 and 40 mg/kg BW ②50 μM for 24 h	↓The expression of serum lipase and amylase ↓Mild edema, inflammatory cell infiltration, and vacuolation of glandular cells ↓Expression of IL-1β, IL-6, and TNF-α ↓Activation of NLRP3 inflammasome ↓NF-κB pathway and ↑Nrf2/HO-1 pathway	Ye et al. (2025)
Kinsenoside	Model: Caerulein plus LPS-induced AP Dose: 2.5, 5, and 10 mg/kg BW	↓Serum amylase, lipase, edema score, inflammation score, necrosis score ↓Number CDD45^+^ cells, macrophage infiltration, M1 macrophage polarization ↓Levels of IL-β and TNF-α ↓TLR4/STAT1 pathway	Wang et al. (2025b)
Bufalin	Model: NaT-induced SAP Dose: 0.1 and 0.2 mg/kg BW	↓Serum amylase and lipase, edema score, inflammation score, necrosis score ↓Serum levels of TNF-α, IL-6, IL-1β, and MDA ↑Serum SOD and GSH ↑Keap1/Nrf2/HO-1 pathway and ↓NF-κB pathway	Niu et al. (2024)
Brusatol	Model: Caerulein-induced SAP Dose: 1.50 mg/kg BW	↓Levels of α-SMA, IL-6, IL-1β, TNF-α, amylase, and lipase ↓NLRP3 inflammasome activation	Zhang et al. (2024a)
Baicalein	Model: NaT-induced SAP Dose: 200 mg/kg BW	↓Serum amylase and pathological score of pancreas and lung ↓Levels of IL-6, IL-1β, TNF-α, and ROS ↓TLR4/NF-κB pathway	Yang et al. (2024)
Saikosaponin D	Model: Caerulein-induced AR42J cells Dose: 10, 20, and 30 μM	↑Cell viability ↓Activities of amylase and lipase, levels of IL-1β, CRP, and IL-18 ↓Oxidative stress and mitochondrial damage ↓NLRP3/caspase-1 pathway	Chen et al. (2024)
Emodin	Model: NaT-induced SAP Dose: 10 mg/kg BW	↓Lipase and amylase levels ↓Pancreas and lung tissue injury ↓CD68^+^ macrophage counts and levels of MPO and TNF-α ↓NF-κB pathway	Hu et al. (2022)
Pachymic acid	Model: Caerulein-induced AP Dose: 20 mg/kg	↓MCP-1 levels, α-SMA, and collagen Ⅰ ↑Pancreas weight ↓Expression of NLRP3, Caspase-1, IL-1β, and IL-18	Li et al. (2022)
Emodin	Model: NaT-induced SAP Dose: 5 and 10 mg/kg BW	↓Pathological score of pancreases and lung ↓Levels of amylase and lipase ↓Levels of IL-6, IL-1β, TNF-α, and MPO ↓Alveolar macrophage pyroptosis and NLRP3-Caspase1-GSDMD pathway	Wu et al. (2022)
Emodin	Model: NaT-induced SAP Dose: 40 mg/kg BW	↓Serum amylase, pathological score of pancreases and lung ↓Activation of the NLRP3 inflammasome and neutrophil recruitment	Jiang et al. (2021)
Borneol	Model: Caerulein-induced AP Dose: 100 and 300 mg/kg BW	↓Pancreas weight, lipase levels, amylase levels, ALT ↓Levels of MDA, MPO, IL-1β, and IL-6 ↓NF-κB pathway	Bansod et al. (2021)
Baicalin	Model: Caerulein-induced AP Dose: 100 mg/kg BW	↓Pancreatic injury and fibrosis, pancreatic stellate cells activation, and macrophage infiltration ↓Expression of Collagen Ⅰ and MCP-1 ↓NF-κB pathway	Fan et al. (2020)
Quercetin	Model ①Caerulein-induced AR42J cells ②Caerulein-induced AP Dose ①10, 20, 30, and 40 μM ②40 mg/kg BW	↑Cell proliferation ↓Levels of TNF-α and IL-6 ↓p38MAPK pathway	Sheng et al. (2021)
Mogroside II_E_	Model ①Caerulein plus LPS-induced AR42J cells ②Caerulein plus LPS-induced SAP Dose ①20 μM ②10 mg/kg BW	↑Cell viability ↓Serum levels of amylase and lipase ↓Levels of TNF-α, IL-6, IL-9, and MCP-1 ↓IL-9/IL-9 receptor pathway	Xiao et al. (2020)
Urolithin A	Model: NaT-induced SAP Dose: 30 mg/kg BW	↓Levels of TNF-α and IL-6 ↓Mitochondrial dysfunction and pancreatic necroptosis	Kang et al. (2024)

Note: NaT, sodium taurocholate; Keap1, Nrf2 and Kelch-like ECH-associated protein 1; Nrf2, Nuclear factor-erythroid 2-related factor 2; TNF-α, tumor necrosis factor-α; COX-2, cyclooxygenase-2; NF-κB, nuclear factor Kappa-B; MCP-1, monocyte chemotactic protein-1.

### 5.1 TCM prescriptions for AP treatment

TCM prescriptions have been utilized in China for preventing and managing AP for a long time. Preclinical studies have identified many TCM formulations with therapeutic efficacy against AP, including *Chaiqin chengqi decoction*, *Dahuangfuzi decoction*, *Chengqi-series decoction*, *Qingyi decoction*, *Qingxiajieyi formula*, *Dachengqi decoction*, *Dachaihu decoction*, and *Chaiqinchengqi decoction* (Yang et al., 2021; Lin et al., 2023; Han et al., 2024; Wen et al., 2024). Moreover, previous studies have demonstrated that AP-associated multi-organ dysfunction (e.g., lung, kidney, liver) can be improved by TCM prescription treatment, such as *Dachengqi decoction* (Liu et al., 2023), *Yinchenhao decoction* (Zhao et al., 2024), and *Chaiqin Chengqi decoction* (Yang X. et al., 2020). Functionally, the main compounds (emodin, rhein, baicalin, and chrysin) from *Chaiqin chengqi decoction* diminished pancreatic acinar cell necrosis and systemic inflammation by inhibiting the TLR4/NLRP3 pathway (Wen et al., 2020). *Qingyi decoction* and its active ingredients (e.g., Wogonoside) alleviated pancreatic injury and systemic inflammation in AP by inactivation of the NF-κB/NLRP3/Caspase-1 pathway (An et al., 2025). Another study by Zhang R. et al. (2024) reported that *Rhizoma Alismatis decoction* alleviated AP by restoration of autophagy flux and mitochondrial homeostasis, leading to downregulation of NLRP3 and IL-1β in the pancreas. Other studies have proved that *Chaiqin Chengqi decoction* (Cao et al., 2024), *Chaihuang Qingfu pill* (Xiao et al., 2025), and *Qingjie Huagong decoction* (Feng et al., 2024) inhibited NLRP3 inflammasome and GSDMD activation-mediated pyroptosis and systemic inflammation in AP models. The above preclinical studies have confirmed the efficacy of TCM prescriptions in treating AP, but are hindered by methodological limitations (e.g., small sample sizes, non-standardized animal models, and lack of randomized controls) and insufficient mechanistic insights (e.g., unclear active ingredients, incomplete safety evaluations, and absence of long-term follow-up data), thus impeding translational validation and clinical implementation.

### 5.2 Herbal extracts in the treatment of AP

In addition to the aforementioned TCM formulas, herbal extracts have shown therapeutic efficacy against AP. For example, free total rhubarb anthraquinones ameliorated intestinal and pancreatic damage in AP rats while reducing inflammation and pyroptosis by inactivation of the NLRP3/caspase-1/GSDMD pathway (Zeng et al., 2024). Meanwhile, rhubarb anthraquinones treatment enhanced intestinal immunity by modulating Treg/Th17 balance (Chen et al., 2016). Xiong et al. (2019) showed that *Lycium barbarum* polysaccharides exhibited anti-inflammatory and antioxidant effects in cerulein-induced AP mice, as evidenced by reduced levels of serum amylase, TNF-α, and IL-1β. Another study reported that *P. guajava* flavonoids reduced pancreatic inflammation and fibrosis by inhibition of NLRP3 inflammasome activation in AP (Zhang G. et al., 2021). Moreover, *Lonicera japonica* flower extracts (Ruan et al., 2019) and *Salvia miltiorrhiza* extracts (Yang et al., 2019) ameliorated AP progression by suppressing the ROS-NLRP3 inflammasome axis and reducing the expression of IL-1β and IL-18. Collectively, these findings highlight the potential of herbal extracts to mitigate AP pathogenesis by targeting the NLRP3 inflammasome and modulating inflammatory cascades.

### 5.3 Monomer components isolated from TCM for the treatment of AP

A wide range of bioactive compounds, including polyphenols, flavonoids, alkaloids, and terpenes, have been isolated and purified from TCM. Pharmacological studies have demonstrated that these TCM-derived compounds exhibited therapeutic potential against AP by suppressing inflammatory response (Lu et al., 2021). The functional roles of these bioactive components in the treatment of AP by targeting NLRP3 are summarized in Table 2.

#### 5.3.1 Polyphenols

Polyphenols, recognized for their anti-inflammatory and antioxidant properties, have shown efficacy in mitigating chronic diseases due to their versatile biological activities (e.g., anti-inflammatory and anti-oxidation) (Vivarelli et al., 2023), which contributed to the treatment of AP. Meanwhile, targeting the inflammasome pathway by polyphenols may be an effective therapeutic strategy for AP (Nani and Tehami, 2023). Resveratrol, a natural polyphenolic compound, has been proven to improve the pathophysiology of AP by reducing inflammation, cell apoptosis, pancreatic damage, blocking calcium overload, and improving microcirculation (Agah et al., 2021; Cai et al., 2025). Recently, resveratrol (Wu S. K. et al., 2024) and epigallocatechin-3-gallate (Luo et al., 2021) exhibited protective effects on severe AP by inactivation of NLRP3 inflammasome. Similarly, paeonol ameliorated AP by promoting M2 macrophage polarization through inactivation of NLRP3 inflammasome (Yuan et al., 2022). Another study found that rosmarinic acid has been shown to reduce inflammation by inhibition of the NF-κB pathway in the murine model of AP (Fan et al., 2015). In addition, scopoletin attenuated AP-induced organ injury (lung and intestine) by blocking the TLR4/NF-κB/NLRP3 pathway (Leema and Tamizhselvi, 2018). Further study highlights the protective effects of urolithin A against AP through suppression of apoptosis and mitochondrial dysfunction (Yang Y. et al., 2023).

#### 5.3.2 Flavonoids

Flavonoids have attracted increasing attention as promising candidates for the modulation of inflammation due to their dual anti-inflammatory and immunomodulatory properties (Al-Khayri et al., 2022). Intriguingly, flavonoids exerted protective effects against AP by targeting key pathogenic processes, including NLRP3 inflammasome activation, oxidative stress, and cytokine storm. For example, baicalein (Wang et al., 2021), rutin (Aruna et al., 2014), naringenin (Li Y. et al., 2018), and luteolin (Rajapriya and Geetha, 2021) have been shown to inhibit the assembly of NLRP3 inflammasome complexes, thereby reducing caspase-1 activation and subsequent IL-1β maturation in AP models. Other studies have proved that proanthocyanidins (Sheng et al., 2023) and baicalein (Wang X. et al., 2025) ameliorated AP by promoting macrophage M2 polarization through suppressing NLRP3 inflammasome activation. Moreover, naringenin improved AP-associated intestinal injury by inhibiting NLRP3 inflammasome activation (Yan et al., 2023). Other flavonoids such as apigenin (Charalabopoulos et al., 2019), biochanin A (Pan et al., 2023), and luteolin (Xiong et al., 2017) alleviated AP by inhibition of the TLR4/NF-κB pathway-mediated inflammation. Zhou et al. (2024) showed that administration of tectoridin inhibited pancreatic injury in AP by triggering macrophage M2 polarization. Another study showed that isorhamnetin alleviated mitochondrial injury and inhibited ROS generation in severe AP (Li X. et al., 2024).

#### 5.3.3 Alkaloids

Alkaloids have emerged as pivotal therapeutic agents in modern medicine due to their broad-spectrum anti-inflammatory and antibacterial properties (Bai et al., 2021). Berberine (BBR), a natural alkaloid extracted from medicinal plants, exhibited multifunctional pharmacological activities (Zhang Y. et al., 2021), including anti-inflammatory, anti-tumor, lipid-lowering, hypoglycemic, and anti-osteoarthritic activities in preclinical studies. Numerous studies have demonstrated that BBR attenuated AP by inhibition of AMPK-mediated inflammation and M2 macrophage polarization (Bansod et al., 2020; Bansod et al., 2025). Meanwhile, BBR treatment improved histological damage to the pancreas, lungs, and intestinal by blocking the NF-κB pathway (Liang et al., 2014; Choi et al., 2017). Li Z. et al. (2020) reported that anisodamine pretreatment mitigated lipopolysaccharide-induced apoptosis and inflammation of pancreatic acinar cells by inactivating the NLRP3 inflammasome and blocking the NF-κB pathway. Moreover, other alkaloids [e.g., castanospermine (Hong et al., 2016), ellipticine (Li X. et al., 2020), rutaecarpine (Huang H. et al., 2021), colchicine (Zhang et al., 2022), matrine (Jin et al., 2023), oxymatrine (Lu M. et al., 2017), nicotine (Zheng et al., 2015)] have been shown to combat AP and AP-induced organ injury by inhibiting the inflammatory response. A recent study showed that galantamine exhibited an anti-inflammatory effect against AP (Thompson et al., 2023), which was an FDA-approved acetylcholinesterase inhibitor for Alzheimer’s disease in clinical trials.

#### 5.3.4 Terpenes

Terpenes represent a structurally diverse class of natural compounds with potent anti-inflammatory and immunomodulatory activities, holding promise for treating inflammation-associated diseases (Chang and Xiong, 2020). Currently, many terpenes (e.g., micheliolide (Wu C. Y. et al., 2024), artesunate (Liu et al., 2025), nimbolide (Bansod and Godugu, 2021), betulinic acid (Zhou et al., 2021), triptolide (Yang et al., 2022), irisin (Han et al., 2023), *etc*.) attenuated AP progression by reducing inflammatory response and inhibiting neutrophil extracellular traps formation. Mechanistically, treatment with ganoderic acid A (Zhang et al., 2025), DGA (Yue et al., 2024), and pachymic acid (Li et al., 2022) improved intestinal dysfunction, macrophage pyroptosis, and pancreatic fibrosis in AP by repressing NLRP3 inflammasome activation. Moreover, other terpenes were found to effectively inhibit necroptosis/apoptosis/ferroptosis and conferr protection against AP, such as celastrol (Liang et al., 2023), crocetin (Zhu and He, 2022), and glycyrrhizin (Cui et al., 2024). Of note, both terpenes [limonin (Xia et al., 2021) and astaxanthin (Kwak et al., 2021)] exert pancreatic protection by suppressing JAK2/STAT3 hyperactivation, thereby reducing pro-inflammatory cytokine production.

#### 5.3.5 Others

Beyond the aforementioned phytochemicals, additional medicinal plant-derived compounds exhibit targeted therapeutic potential against AP. Cordycepin, a nucleoside derivative extracted from *Cordyceps militaris*, inhibited pancreatic inflammation and injury by blocking the NF-κB/NLRP3 inflammasome pathway (Yang J. et al., 2020). Anthraquinones (e.g., emodin) exerted pleiotropic effects on inflammation and pancreatic tissue repair via the inactivation of NLRP3 inflammasome (Zhang et al., 2019). Shikonin (Xiong et al., 2013) and Astragalus polysaccharides (Wang Q. et al., 2025) mitigated AP progression by inhibiting NF-κB pathway-mediated inflammation. Moreover, the protective effect of notoginsenoside R1 (He et al., 2022) and protocatechuic acid (Abdelmageed et al., 2021) on AP-induced lung injury by blocking the HMGB1/TLR4/NF-κB pathway. Collectively, these findings underscored that bioactive compounds derived from TCM counteract AP pathogenesis by targeting inflammatory pathways, including NF-κB, MAPK, and NLRP3 inflammasome.

## 6 Clinical study of TCM for the prevention and treatment of AP

Preclinical studies have confirmed that TCM possesses significant therapeutic potential against AP. Notably, randomized controlled trials have revealed that TCM interventions reduced mortality rates, shortened hospitalization duration, and mitigated postoperative complications in AP patients compared to conventional therapies (Qiong et al., 2005). For instance, among the included 248 patients with AP (124 patients in each group), *Chaiqinchengqi decoction* treatment reduced the duration of 28-day respiratory failure (median: 1.0 days, 95% confidence interval: −2.0 to 0.0) and improve 6-month clinical outcomes in AP patients compared with the placebo (Deng et al., 2025). Similarly, *Dachengqi decoction*, as adjunctive therapy, reduced multiple organ dysfunction syndrome incidence by 40% and pancreatic infection risk by 5% in severe AP patients (Chen et al., 2010). *Tongfu powder* treatment alleviated gastrointestinal dysfunction in AP cohorts (Miao et al., 2018). Moreover, integrative approaches combining TCM with Western medicine exhibited synergistic benefits, including decreased organ failure risk (4.1% *vs*. 5.9%), reduced hospitalization costs ($2,157/patient *vs*. $2,895/patient) and overall mortality rate (1.7% *vs*. 3.4%) (Deng et al., 2024). Notably, *Guo qing yi tang decoction* treatment enhanced intestinal barrier integrity (serum DAO and MFG-8↓, p < 0.05) and reduced inflammatory cytokines (TNF-α, IL-6, and IL-8↓, p < 0.05), APACHE II scores (7.84, p < 0.001), and hospital stay after 1 week in a total of 38 AP patients compared with the control group (cluster therapy alone, 70 patients) (Chen et al., 2021). These findings contrast with the limitations of conventional Western medications, including high costs and adverse effects. Herein, ongoing national clinical trials evaluating TCM safety and efficacy in AP are summarized in Table 3. Meanwhile, some preclinical and clinical studies have proved that no significant adverse effects were observed during the TCM treatment period (Wan et al., 2011; Lu et al., 2014). However, TCM formulas may cause mild gastrointestinal discomfort, such as nausea or diarrhea (Tan et al., 2020). Other TCM herbs may interact with antiplatelet or anticoagulant drugs, increasing the risk of bleeding (Li et al., 2019b).

**TABLE 3:** Clinical trials of TCM in AP.

Category	Year of registration	Enrollment	Sponsor	Recruiting status	Clinical trial ID
*Dachaihu decoction*	2023	306	Fifth Affiliated Hospital, Sun Yat-Sen University, China	Not yet recruiting	NCT04990336
*Qingyi granule*	2007	300	Tianjin Nankai Hospital, China	Unknown status	NCT00508729
*Qingyi Jiangzhi decoction*	2024	100	The First People’s Hospital of Lianyungang, China	Not yet recruiting	ChiCTR2400094917
*Liuhe Dan ointment*	2024	240	West China Hospital of Sichuan University, China	Not yet recruiting	ChiCTR2400085136
*Qingyi Jiangzhi decoction*	2024	100	The First People’s Hospital of Lianyungang, China	Not yet recruiting	ChiCTR2400094917
*Daxianxiong decoction*	2024	108	Chongqing Hospital of Traditional Chinese Medicine, China	Recruiting	ChiCTR2300076885
*Qing Yi Dao Xie decoction*	2023	90	Changsha Hospital of Traditional Chinese Medicine (Changsha Eighth Hospital), China	Recruiting	ChiCTR2300078065
*Qingyi granule*	2022	340	The First Affiliated Hospital of Dalian Medical University, China	Recruiting	ChiCTR2200061929
*Rhubarb decoction*	2021	112	The First Affiliated Hospital of Chongqing Medical University, China	Recruiting	ChiCTR2100046548
*Chaiqin Chengqi decoction*	2020	248	West China Hospital of Sichuan University, China	Completed	ChiCTR2000034325
*Qing Yi decoction*	2015	120	The First Affiliated Hospital of Guangxi Medical University, China	Completed	ChiCTR-OIR-15007512
*Radix paeoniae* rubra	2014	60	Shanghai Changhai Hospital, China	Completed	ChiCTR-TRC-14004664
Emodin	2014	250	First Affiliated Hospital, Dalian Medical University, China	Completed	ChiCTR-TRC-14004653
Rhubarb	2013	300	The first affiliated hospital of Nanchang University, China	Completed	ChiCTR-TRC-13003573
*Da-Cheng-Qi decoction*	2012	21	The First Affiliated Hospital of Wenzhou Medical University, China	Completed	ChiCTR-ONRC-12002792

Despite these promising results, translating preclinical TCM research into clinical practice faces multifaceted challenges. A primary hurdle involves securing regulatory approval for commercialization due to resource limitations and difficulties in semi-synthetic production or medicinal plant engineering of bioactive compounds. This dependency on natural resources raises ethical concerns, particularly given reports of over 20,000 medicinal plant species at risk of extinction. Additionally, critical gaps remain in mechanistic understanding and robust clinical data. Other challenges include poor solubility/absorption profiles, intellectual property issues, and limitations in drug-likeness and purity of TCM-derived compounds. Of note, integrating TCM into standard care was constrained by methodological limitations (e.g., small sample sizes, non-RCT designs), mechanistic ambiguity, and quality control issues, necessitating large-scale randomized trials, ingredient standardization, and safety evaluations to facilitate evidence-based implementation.

## 7 Conclusion and perspectives

Recent advances in elucidating the pathogenesis of AP have coincided with the growing recognition of TCM as a promising therapeutic strategy. Accumulating evidence highlights TCM’s remarkable therapeutic efficacy in AP management through multi-target modulation, particularly its capacity to regulate NLRP3 inflammasome activation and downstream inflammatory cascades. This review systematically summarized current knowledge on TCM-derived compounds targeting NLRP3-mediated pathways in AP, while critically addressing persisting challenges in translational applications. Key limitations hindering clinical translation include: (1) *Formula standardization gaps*: Current research predominantly focuses on empirical or self-formulated TCM prescriptions, with insufficient validation of classical prescriptions through randomized controlled trials; (2) *Bioavailability challenges*: Many active ingredients of TCM exhibited suboptimal pharmacokinetic profiles due to structural instability, rapid oxidation, and poor membrane permeability, advanced delivery systems (e.g., lipid-based encapsulation, nanoparticle carriers) can be considered; (3) *Safety and metabolism uncertainties*: Comprehensive characterization of TCM pharmacokinetics, tissue distribution, and long-term toxicity in AP-specific contexts remains imperative; (4) *Mechanistic complexity*: The polypharmacological nature of TCM necessitates integrative multi-omics approaches, including metabolomics, network pharmacology, proteomics, immunomics, and gut metagenomics, to decode its regulatory effects on NLRP3 inflammasome; (5) Standardized protocols in TCM research are essential to ensure reproducibility, validate therapeutic efficacy, and facilitate regulatory acceptance for broader clinical implementation.

In summary, NLRP3 inflammasome inhibition represented a strategic diagnostic and therapeutic nexus in AP. TCM served as a reservoir of NLRP3 inflammasome-modulating agents with the potential to restrict AP progression. Of note, this review provided a framework for evidence-based optimization of TCM to combat AP, advocating for the integration of systems biology and advanced drug delivery platforms to bridge traditional knowledge with modern precision medicine paradigms.
